# Strengthening health systems capacity to monitor and evaluate programmes targeted at reducing abortion-related maternal mortality in Jessore district, Bangladesh

**DOI:** 10.1186/s12913-015-1115-6

**Published:** 2015-09-28

**Authors:** Fauzia Akhter Huda, Anisuddin Ahmed, Evelyn Rebecca Ford, Heidi Bart Johnston

**Affiliations:** Centre for Reproductive Health, icddr,b, GPO Box 128, Dhaka, 1000 Bangladesh; Oregon Health & Science University School of Medicine, Portland, OR USA; World Health Organization, Geneva, Switzerland

**Keywords:** Health system, Menstrual regulation, Abortion, Post abortion care, Maternal mortality, Bangladesh

## Abstract

**Background:**

Abortion related deaths as a proportion of maternal mortality appears to have fallen dramatically in Bangladesh from 5 % in 2001 to 1 % in 2010. Yet complications from menstrual regulation (MR) and unsafe abortion continue to cause deleterious health, economic and social consequences for women in the country.

**Methods:**

This quasi experimental design study with a baseline (January to December 2008) and an endline survey (August to October 2009) was conducted in 69 public, private, and NGO sector health facilities in Jessore district of Bangladesh with the objective of adapting and implementing a set of process indicators, specifically to supplement the indicators for monitoring emergency obstetric care interventions. At the baseline, we collected retrospective data from all 69 health facilities that provided MR, legal abortion or post-abortion care (PAC), by reviewing their last one year’s records. Three months after introducing the safe menstrual regulation and abortion care (SMRAC) model, endline data was collected. Signal function (critical services that facilities must perform in order to prevent and treat abortion complications) analysis was used to characterize facilities as providing basic care, comprehensive care, or neither. Facility mapping, and records on services provided and complications treated were used to further characterize service availability and to describe service use and quality.

**Results:**

No facilities fulfilled criteria for ‘comprehensive’ care at either the baseline or end line while only one met the ‘basic’ criteria during the endline of the project. Recommended uterine evacuation technology, manual vacuum aspiration (MVA) was used for 100.0 % of MR clients but only for 8.0 % or fewer PAC patients. MR clients were 37.5 times more likely than PAC patients to leave facilities with a contraceptive method (75.0 % vs. 2.0 %).

**Conclusion:**

Persistent use of older uterine evacuation technologies was observed when recommended techniques were widely available in the facilities. Notable gaps were identified in providing post-abortion contraceptive services for women treated for PAC. By systematic implementation of the SMRAC model, health systems can track and measure progress and gaps in their implementation and identify strategies for further reduction of abortion-related morbidity and mortality in Bangladesh.

## Background

Globally, slow progress has been made toward Millennium Development Goal 5 (MDG 5) of reducing maternal mortality ratio by 75.0 % between 1990 and 2015. Many countries, particularly those with low resources, have not met the necessary 5.5 % annual decline necessary to be on track for the 2015 deadline [1]. Nonetheless, in Bangladesh, maternal mortality ratio has decreased by 66.0 % in the last 20 years, from an estimated 574 to 194 per 100,000 live births between 1990 and 2010 [2]. The annual abortion-related deaths per 100,000 women of reproductive age decreased by 87.0 % from an average of 17 in the years 1976–1985 to an average of 2 for the years 1996–2005 in Matlab, a rural area of Bangladesh [3]. The Bangladesh Maternal Mortality Surveys from 2001 to 2010 recorded that abortion-related mortality as a percent of all maternal deaths fell from 5 to 1 % [2]. Longitudinal data suggest that reductions in abortion-related mortality – a cause of 13 % of maternal mortality globally – may have contributed to this decline [4]. With its current annual rate of decline in maternal mortality of 5.5 %, Bangladesh appears to be one of the few countries on track to meet the primary target of MDG 5 [2]. Yet women who do not want to become pregnant and do not have access to contraception are at risk of unplanned pregnancy and unsafe abortion [5]. This result in complications arising from MR procedures and unsafe abortion continue to cause deleterious health, economic and social consequences not only for the women but also for the society as a whole, both in the short term and in the long run [6].

Abortion is illegal in Bangladesh except only to save the life of the woman. Since the 1970s, Bangladesh has maintained a menstrual regulation (MR) program, which is defined as an interim method of establishing non-pregnancy in women at risk of being pregnant [7]. MR has an advantage in countries where abortion is legally banned because it can be conducted without a confirmatory pregnancy test, within 10 weeks of the beginning of the last menstrual period by family welfare visitors (health service providers having at least ten years of formal schooling and 18 months of training in family planning and maternal and child health care, and additional training specifically in MR) and within 12 weeks of a missed menstrual period by medical doctors [8, 9]. Although MR services have been decentralized, estimates showed that the incidence of induced abortion was the same as that of MR in 2010, (647,000 and 653,000 respectively) which implies that the demand for MR services are not being met [10]. Also there were 231,400 women treated for complications of induced abortions in the same year and another 341,000 complicated cases did not get any medical care [10]. Additionally, an estimated 78,000 complications from MR were found which indicate that the quality of clinical services needs to be strengthened [10].

Over the last decade, a set of tools and indicators have been used to monitor emergency obstetric care (EmOC) interventions for reducing maternal deaths. These UN EmOC indicators include treatment of abortion complications [5, 11]. However, essential preventive means to reduce unsafe abortion are not represented. A recent study in Bangladesh showed that in the public sector, a system for collecting data on MR and PAC exists, but underreporting is common, while in the private sector, there is limited or no data [6]. Although this study gave estimates for MR and PAC in one point in time, it pointed out that having consistent and comparable data on a continuous basis would help the government in identifying gaps in the health system that influence provision of MR and abortion for legal indications [6].

As for the health system in Bangladesh, in the public sector, the largest health service provider of the country is the Ministry of Health & Family Welfare (MoH&FW). There are two major implementation wings under the MoH&FW- the Directorate General of Health Services (DGHS) and the Directorate General of Family Planning (DGFP). The DGHS is responsible for implementation of all public health programmes in the country including emergency preparedness and response (EPR) programme. The DGFP is responsible for implementing family planning (FP) programmes and providing FP related technical assistance to the MoH&FW [12, 13].

To address the gap on consistent and continuous data on MR and PAC, we conducted this study to adapt and implement a set of process indicators, based on the safe abortion care (SAC) model, developed by Ipas, a reproductive rights non-profit organization based in the US, specifically to supplement the tools and indicators for monitoring emergency obstetric care interventions. In this study, The SAC model was validated and adapted as the safe menstrual regulation and abortion care (SMRAC) model [9, 14] with the objective of assessing the feasibility, acceptability and utility of the SMRAC model in Jessore district of Bangladesh. We also documented the availability, utilization, and quality of MR and abortion services over time, and highlighted elements of care in need of quality improvement.

## Methods

This quasi experimental design study was conducted in Jessore, a district in the southwest part of the country, with a baseline (January to December 2008) and an endline survey (August to October 2009). All 74 registered health facilities [public (*n* = 44), private-for-profit (*n* = 22), and NGO not-for-profit (*n* = 8)] in Jessore, that provided MR, legal abortion, and/or PAC services were enlisted in the project. Among the 44 public sector facilities 36 were under the management of the Directorate General of Family Planning (DGFP) and eight were under the Directorate General of Health Services (DGHS) of the Ministry of Health & Family Welfare (MoH&FW). Two of the public sector facilities including district hospital and maternal and child welfare centre (MCWC), situated at district level, provided secondary level of health care and the remaining 42 facilities including upazilla health complexes (UHC) placed at upazilla/sub-district level, union health and family welfare centres (UH&FWC) and family welfare centres (FWC) placed at union level, provided primary level of health care.

The SMRAC model focuses on three service areas essential for reducing abortion-related maternal mortality: MR and abortion for legal indications, treatment for abortion complications (complications of spontaneous abortion and MR as well as complications of unsafe induced abortion), and contraceptive services for MR and PAC patients. The SMRAC model comprises of seven indicators and several signal functions (defined as critical services that facilities must provide to prevent and treat complications due to MR & abortion).

We assessed availability and distribution of SMRAC services by determining the 10 signal function performance at each facility (listed in Table 1). Facilities that offered all 6 basic signal functions in the previous 3 months were considered as “basic” and facilities that performed all 10 signal functions were considered as “comprehensive.” The 6 basic signal functions are attainable for health centers and include services for first trimester induced abortion and treatment of complications. The additional 4 comprehensive signal functions are more consistent with hospitals’ capacity and include surgery and blood transfusion as well as second trimester abortion care. The SMRAC indicators (listed and defined in Table 2) were used to monitor facility availability, distribution, service utilization, and quality of care [5, 9, 14].

**Table 1:** Signal functions for safe menstrual regulation and abortion care (SMRAC)

Signal functions for basic SMRAC services	Signal functions for comprehensive SMRAC services
● Administer essential antibiotics	*Perform all basic functions plus*
● Administer intravenous fluids	
● Administer oxytocics	● Perform safe, legal abortion for uterine size >12 weeks, for all legal indications
● Perform removal of retained products for uterine size ≤12 weeks	● Perform removal of retained products for uterine size >12 weeks
● Perform MR for uterine size ≤10 weeks and/or safe, legal abortion (≤12 weeks)	● Perform blood transfusion
● Provide post-MR and post-abortion contraception	● Perform laparotomy

Table adapted from Healy et al. [9], originally adapted from UN guidelines (Maine et al. [14])

**Table 2:** Seven Indicators for measuring SMRAC

SMRAC indicators	Definition	Recommendation
*Are enough facilities providing SMRAC services?*		
1. Amount of SMRAC services available	Number of facilities providing basic and comprehensive SMRAC	For every 500,000 population: 5 SMRAC facilities, at least 1 of which offers comprehensive SMRAC
*Are SMRAC services well-distributed?*		
2. Distribution of SMRAC facilities	Number of facilities providing basic and comprehensive SMRAC in sub national areas	Minimum: 100 % of sub-national areas have adequate level of SMRAC as recommended in indicator 1
*What proportion of services for women with obstetric complications are services for MR/abortion complications?*		
3. Proportion of women treated for obstetric complications that are MR/abortion related	Numerator: number of women with MR/abortion complications treated at a facility in a given period;	Over time, a declining percentage of women with abortion/MR complications
	Denominator: number of all women with obstetric complications treated at facility in the same time period	
*How common are serious abortion/MR complications?*		
4. Proportion of women treated for MR/abortion complications that are serious	Numerator: number of women with serious MR/abortion complications treated at a facility in a given period; Denominator: number of all women with any MR/abortion complications treated at facility in the same time period	Over time a declining percentage of women with serious MR/abortion complications
*To what extent are MR and safe, legal abortion being provided?*		
5. Proportion of women who receive MR and safe, legal abortion among all women receiving MR/abortion care	Numerator: number of women receiving MR and safe, legal abortion procedures at a facility in a given time period	Over time, a shift towards a higher proportion of women receiving MR and safe, legal abortion
	Denominator: number of all women receiving MR and abortion-related services in facility in the same time period	Recommended level: Approaching 100 %
*Are appropriate technologies being used?*		
6. Proportion of uterine evacuations performed with appropriate technology	Numerator: number of uterine evacuation procedures performed with appropriate technology at facility in a given period.	Over time, a shift toward a higher proportion of procedures performed with appropriate technology as per WHO recommendations^a^
	Denominator: number of all uterine evacuation procedures performed at facility within the same time period	Recommended level: 100 %
*Are women who have received MR or safe, legal abortion or treatment for MR/abortion complications provided contraception before being discharged from a facility?*		
7. Proportion of women receiving MR, safe, legal abortion, or treatment for MR/abortion complications who obtain contraception before leaving facility	Numerator: number of women receiving MR, safe, legal abortion or treatment for MR/abortion complications who obtain contraception before leaving facility	At least 60 % of all women receiving MR/abortion services accept contraception
	Denominator: number of women receiving MR, safe, legal abortion, or treatment for MR/abortion complications in facility in the same time period.	

Adapted from Healy et al. [9]

^a^WHO has provided evidence-based guidance on the preferred methods of uterine evacuation for different stages of pregnancy [17]

### Data collection

The project was implemented in five phases:

***Phase 1:*** Development and adaptation of study materials to be appropriate to the country setting; reviewed and revised materials based on stakeholder recommendations; pretested study tools; revised the baseline and monitoring tools based on pretest results. (January-February)

***Phase 2:*** Baseline data was collected from facility record book for the period January-December 2008 (secondary data). Data were analyzed and preliminary results were shared with policy makers and representatives of participating facilities at an orientation workshop. (March-April)

***Phase 3:*** Training of staffs from participating facilities on the new SMRAC model and introduction of the tool in all selected facilities. Regular visit of project staff to the facilities for monitoring and helping the service providers to use the SMRAC model (May-July 2009).

***Phase 4:*** Endline data collection by project staff (August-October 2009) from the new SMRAC model (primary data).

***Phase 5:*** Comparative data analysis of baseline and endline data; findings shared to local and national stakeholders from the public, private, and NGO health facilities.

### Data analysis

Data were entered into Microsoft Excel 2007 and cross-checked against original entry forms. All data were converted into IBM SPSS (20 version) for analysis. The SMRAC indicators were calculated for 2 periods: a year‐long baseline data (January to December 2008) and endline data (August-October 2009). Results were reported for each SMRAC indicator. Signal function analysis was done to characterize facilities as providing basic care, comprehensive care, or neither. Facility mapping, and records on services provided and complications treated were analyzed to further characterize service availability and to describe service use and quality. The Chi-square test was used to compare baseline and endline indicators for private, public, and NGO sector health facilities. Significance was evaluated at *P* < 0.05.

### Ethical approval

The study was approved by the Institutional review board (IRB) of International Centre for Diarrhoeal Disease Research, Bangladesh (icddr,b).

## Results

We report findings from the implementation of the seven SMRAC indicators, first in terms of availability and distribution of services and then in terms of use and quality of services. Among the 74 participating facilities, five facilities (4 private and 1 NGO) dropped out before project completion. Owners of the four private facilities felt that participation in the project was time-consuming and not beneficial to their business and the NGO clinic had closed due to lack of funds. Finally, at the endline, data from 69 health facilities has been presented.

### Availability and distribution of SMRAC services

#### Indicator 1. Are enough facilities providing SMRAC services?

With a population of 2,469,680 [15], Jessore is recommended to have a minimum of 20 basic facilities and five comprehensive facilities. From all the participating facilities, only one private hospital met the basic SMRAC criteria during the endline data collection period and none of the facilities met the criteria to perform all ten signal functions in the endline (Table 3). Four facilities reported to providing eight to nine signal functions. One of these facilities—the district hospital—was documented as providing eight of the ten signal functions. Each of the individual basic signal functions was offered at a minimum of 25 facilities. Three of the four comprehensive signal functions were offered at 19 or more facilities though performance of safe, legal abortion for gestations over 10 weeks was recorded at a maximum of only 4 facilities (Table 3).

**Table 3:** Signal functions provided by health facilities in the study area

Signal function	Baseline						Endline					
	(*n* = 74)						(*n* = 69)					
	FWC	MCWC	UHC	Dist. Hosp.	Private	Total	FWC	MCWC	UHC	Dist. Hosp.	Private	Total
	*N* = 30	*N* = 1	*N* = 12	*N* = 1	*N* = 30	*N* = 74	*N* = 29	*N* = 1	*N* = 12	*N* = 1	*N* = 26	*N* = 69
Administration of antibiotic	0	1	7	1	21	30	0	1	7	1	20	29
Administration of intravenous fluid	0	1	7	1	22	31	1	1	7	1	19	29
Administration of uterotonics	0	1	7	1	23	32	0	1	7	1	20	29
Removal of retained products (≤12 weeks)	0	0	8	1	18	27	0	0	7	1	18	26
Post-procedure contraception	23	1	4	0	4	32	24	1	5	0	6	36
MR (≤10 weeks) and/or safe, legal abortion (≤12 weeks)	28	1	4	0	6	39	25	1	5	0	6	37
Basic SMRAC facilities (20 recommended: 4 per 500,000 population)	0	0	0	0	0	0	0	0	0	0	1	1
Removal of retained products (>12 weeks)	0	0	6	1	13	20	0	0	6	1	15	22
Blood transfusion	0	0	4	1	18	23	0	0	4	1	19	24
Laparotomy	0	0	2	1	18	21	0	0	2	1	16	19
Safe, legal abortion (>12 weeks)	0	0	0	0	1	1	0	0	2	1	1	4
Comprehensive SMRAC facilities (5 recommended:1 per 500,000 population)	0	0	0	0	0	0	0	0	0	0	0	0

#### Indicator 2. Are SMRAC facilities well distributed?

**Fig. 1:**
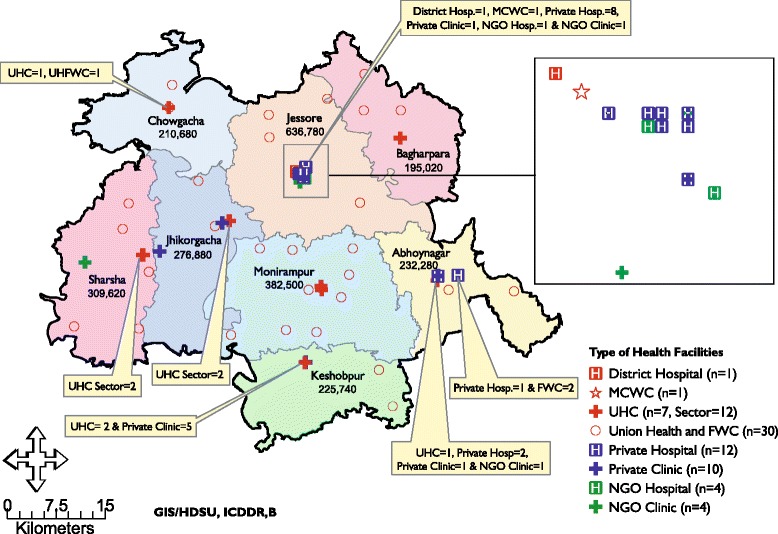
Distribution of health facilities in study area, Jessore-2009

Jessore is divided into 8 upazilas/sub-districts; the district hospital is located in the *sadar* (town center) upazila. The other seven upazilas has multi-bedded UHCs; five of which has both functioning health and FP wing, whereas two of the UHCs (Chowgacha and Bagharpara) have only the health wing (no FP wing). The 30 FWCs and UH&FWCs are well-distributed throughout the district and provide MR and contraceptive services. Private and NGO health facilities were scattered less evenly throughout the study area (Fig. 1).

### Use of SMRAC services

#### Indicator 3. What proportion of services for women with obstetric complications are services for MR/abortion complications?

Over the SMRAC project period, within a month, an average of 179 women was admitted with obstetric complications that were originally caused due to MR/abortion complications. At baseline, 51 % of the obstetric complications were identified as abortion-related which decrease to 29 % at the endline reflects a reduction in the abortion complication caseload and a substantial increase in the total obstetric complication caseload (Table 5). This was due to proper categorization of the cases and accurate record keeping using the SMRAC model. Overall, the proportion of abortion cases among all obstetric complications was higher in private facilities than NGO or public facilities (Table 4).

**Table 4:** Safe menstrual regulation and abortion care indicators (Indicators 3–7)

Indicator 3	Baseline	End line	Total	*p-*value
Abortion-related complications as a percentage of obstetric complications	(Jan to Dec 2008)	(Aug to Oct 2009)		(sig. in change from baseline to endline)
NGO (%)	54.12	26.42	45.66	0.000
	368/680	79/299	447/979	
Private (%)	68.08	36.30	57.00	0.000
	561/824	200/551	761/1335	
Public (%)	44.16	24.56	38.79	0.000
	1062/2405	223/908	1285/3313	
Indicator 4				
Serious abortion-related complications as a percentage of all abortion-related complications by type of facility				
NGO (%)	0.27	10.13	2.01	0.000
	1/368	8/79	9/447	
Private (%)	23.53	9.00	19.71	0.000
	132/561	18/200	150/761	
Public (%)	11.21	13.00	11.52	0.4220
	119/1062	29/223	148/1285	
Indicator 5				
Proportion of all women receiving abortion services who receive MR or safe, legal abortions				
NGO (%)	92.92	92.94	92.92	0.990
	4829/5197	1040/1119	5869/6316	
Private (%)	1.23	33.33	12.33	0.000
	7/568	100/300	107/868	
Public (%)	57.47	68.94	60.03	0.000
	1435/2497	495/718	1930/3215	
Indicator 6				
Proportion of uterine evacuation procedures performed with recommended technology				
NGO (%)	95.36	94.93	95.28	0.531
	4829/5064	1048/1104	5877/6168	
Private (%)	4.66	33.67	14.56	0.000
	27/579	101/300	128/879	
Public (%)	67.17	67.36	67.22	0.964
	1543/2297	487/723	2030/3020	
Indicator 7				
Proportion of women receiving MR or abortion services who obtained contraception before leaving the facility				
NGO (%)	58.40	89.10	63.84	0.000
	3035/5197	997/1119	4032/6316	
Private (%)	0.00	29.67	10.25	0.000
	0/568	89/300	89/868	
Public (%)	45.33	58.22	48.21	0.000
	1132/2497	418/718	1550/3215	

#### Indicator 4. How common are MR/abortion related severe complications?

Severe complications are defined as those that are or can quickly become life-threatening if not treated immediately and includes shock, severe vaginal bleeding, intra-abdominal injury, and sepsis [16, 17]. Over the two periods of measurement, data for the indicator did not demonstrate any trend. During the baseline 13 % of all cases with MR/abortion complications were considered severe, while during endline, the percentage went down to 11 % (Table 4). Death due to severe complications was not captured in the study.

#### Indicator 5. To what extent are MR and safe, legal abortion being provided?

Findings from the study showed that MR and safe, legal abortion (abortion that needs to be done to save the life of the woman) made up the majority (75–77 %) of abortion-related care (Table 4). NGOs performed the majority of safe MR procedures (93 % in the endline) among women receiving abortion services, followed by public facilities (69 %) and private facilities (33 %) (Table 4). Only 8 legal abortions were recorded during the project period. No temporal trends in this indicator were detected.

### Quality of SMRAC services

#### Indicator 6. Are appropriate technologies being used?

Manual vacuum aspiration (MVA), a safe and effective method of abortion that involves evacuation of the uterine contents by the use of a hand-held plastic aspirator. It is appropriate for treatment of incomplete abortion for uterine sizes up to 12 weeks from the last menstrual period (including miscarriage, spontaneous abortion and removal of retained products from an induced abortion), first-trimester abortion (menstrual regulation) and endometrial biopsy. Compared to the sharp-curettage method of abortion, use of MVA requires less cervical dilatation and is associated with less blood loss, shorter hospital stays and a reduced need for anesthetic drugs. Vacuum aspiration methods (either manual or electric vacuum aspiration) are recommended over sharp curettage by the World Health Organization [18]. Considering all abortion-related uterine evacuations, the recommended technology, MVA was used 77–81 % of the time. A disparity in the appropriateness of technology used was revealed between MR care and PAC services. As shown in Table 5, recommended technology was used for all the MRs performed over the study periods (baseline and endline). In stark contrast, only 8 % of the abortion complication patients were treated with appropriate technology (MVA) in the baseline and less than 1 % in the endline; the remaining were mostly treated with dilatation & curettage (D&C). By the endline, majority of the NGOs (94 %) were providing uterine evacuation with recommended technology compared to public (67 %) and private (33.3 %) facilities (Table 4).

**Table 5:** Contraceptive acceptance following MR and PAC during the project period

Case definition	Number of patients /clients	Received FP methods^a^		Received FP referral^b^		Total receiving FP referral or supplies	
		No.	%	No.	%	No.	%
Care for MR	9671	7233	75	26	0.3	7259	75
Care for PAC	3067	69	2	405	13	474	15

^a^Defined as leaving the facility with a contraceptive method

^b^Defined as referred to another facility for contraceptive services

#### Indicator 7. Are women who have received MR, safe legal abortion or treatment for abortion complications provided contraception before discharge?

The aggregated data show that at the beginning of the SMRAC project only 50 % of MR clients and women treated for abortion-related complications were recorded as having received contraceptive services (Table 4). Over the course of the project this increased to 70 %. A closer look at the data showed that only nine of 69 facilities reported consistent increases in provision of post-MR or post-abortion contraceptive services. By the endline, NGO facilities provided women with more contraceptives (89 %) than public (58 %) and private (29.7 %) facilities (Table 4).

Over the data collection periods, 75 % of MR clients were recorded as having left the facility with a contraceptive method, whereas only 2 % of PAC patients were recorded as acceptor of a contraceptive method; MR clients were 37.5 times more likely than PAC patients to leave facilities with a contraceptive method (75 % vs. 2 %) (Table 5). An additional 13 % of PAC patients were referred to another facility for contraceptive services.

## Discussion

Implementation of the SMRAC model in Jessore has provided evidence that to meet the SMRAC recommendations, the district needs more facilities that offer six basic and ten comprehensive signal functions. However, it was observed that elements of services were more available than the SMRAC basic and comprehensive signal function indicators suggest. The current distribution of health facilities is appropriate, but many require improvements in delivering basic and comprehensive care. Results from the implementation of indicator one suggested that Jessore needs 19 additional basic SMRAC facilities and five comprehensive SMRAC facilities.

Based on the country’s existing health system, post abortion care (PAC) services are offered in the health wing of the Upazila Health Complex (UHC) and referred to the family planning (FP) wing of the same health complex for contraceptive services. As a result, the health wing of each UHC consistently provided the first four of the six basic signal functions, and the FP wing consistently provided the remaining two: MR and post-MR/PAC contraceptive counseling and services. There is no system of follow-up in the health wing to track contraceptive acceptance among this group of patients, as the FP wing maintains separate records. If there was no administrative division between the health and family planning wings, five UHCs likely could become comprehensive SMRAC facilities.

The present study showed that, only one private hospital met the basic SMRAC criteria during the endline data collection period and none of the facilities met the comprehensive SMRAC criteria either in the baseline or endline. One of the facilities, the district hospital was found as provider of the eight signal functions. The two missing signal functions were: contraceptive acceptance for PAC patients and MR services delivery with associated contraceptive services. Both of these services were provided by one NGO, RHSTEP (Reproductive Health Services Training and Education Program, the only NGO which is permitted by the Government of Bangladesh to perform its activities in 13 public medical college hospitals and nine district hospitals) [19] on the district hospital campus and appear as services provided by the NGO sector; even though the PAC patients were referred to RHSTEP from the district hospital. Additionally, if staff at the union level, who are assigned to provide MR and post-MR contraception could be simultaneously trained, equipped and authorized to provide antibiotics, IV fluids, and uterotonics, to remove retained products and to refer clients with severe complications to the appropriate level of care, then 30 well-distributed FWCs and UH&FWCs of the study area could become basic SMRAC facilities.

Though MR and PAC services were found available in Jessore but MR care meets quality indicators than the PAC services. Disaggregation of contraception provision data by MR clients and PAC patients revealed another disparity in the quality of care received by these two groups. A large number of MR clients (75 %) left the facility with a contraceptive method; while only 2 % of the PAC patients received any contraceptive method during leaving the facility and another 13 % were referred to other facility for contraceptive services. It is possible that additional patients were referred for or supplied with a family planning method without a record of that referral.

The SMRAC model helped in identifying important gaps in the care women receive, notably the absence of contraceptive services for women treated for PAC and the persistent use of D&C for uterine evacuation when the recommended improved technologies (MVA) are widely available in Bangladesh. The striking difference in technology used observed in this study implies a role for targeted capacity building at the facilities. The high numbers of abortion complications identified indicates a need to explore means of reducing unwanted pregnancies and unsafe abortions by increasing access to contraception and MR. Implementation of the SMRAC model also demonstrated the benefits of improved reporting by highlighting areas in need of service delivery improvement.

Findings showed that uptake and continued use of SMRAC model by the majority of facilities was encouraging. Although the research was an attempt to measure the comprehensiveness and quality of the care available in the area, it implicitly assumes that monitoring will result in improvements in services, and the analysis attempts to identify any such impacts. While many providers expressed positive feedback for the SMRAC model and its impact on their record-keeping– including several WHO attributed enhanced capacity for monitoring, evaluation, performance, and quality enhancement through the model, but some expressed inability to use the model due to limits on time or excessive pre-existing workload. Several private clinics expressed concern that use of the model would not prove beneficial to their business or profit. Many providers suggested integration of SMRAC models with government-led monitoring and evaluation.

### Study limitations

The study has several limitations. First, there is the possibility that data were misclassified during the early stages of SMRAC implementation. Several dramatic shifts were observed in reported service delivery during the project period. These shifts may have more to do with increased familiarity with the SMRAC data collection process than with changes in service delivery. Second, the SMRAC indicators were developed for efficient implementation. The quality of care indicators have the advantage of ready quantifiability, however they are not complete measures of quality of care. For example, they do not include aspects of care such as client-provider interaction, level of skills that the providers possess, or the proportion of women who were rejected from MR services which can substantially influence accessibility of care and service delivery equity. These indicators can be included in the revised SMRAC model to better capture the quality of care issues. Third, the monitoring mechanisms were in effect for too short a time to expect much change in the facilities, and the baseline data were not comparable to the monitoring data in some respects. Additionally, there was no comparison group of health facilities in which the SMRAC model was not being implemented. Furthermore, conducting the study in only one district may have made the findings less generalizable.

## Conclusion

Globally unsafe abortion is a leading contributor to maternal morbidity and mortality even though means of prevention are well known and accessible. Data suggest that Bangladesh has made progress in reducing maternal mortality due to unsafe abortion and increased access to MR along with emergency obstetric care has been shown to contribute to this reduction [20]. Implementation of the SMRAC model has provided details of the health system strategies in place that likely have contributed to this achievement. Additionally the SMRAC model has highlighted gaps that if addressed could yield further reductions in abortion-related morbidity and mortality. Hence monitoring the availability, utilization, and quality of MR and PAC services using the SMRAC model will help identify whether these services are reaching recommended standards so that further reductions in the MMR can be achieved.

### Recommendations

Analysis from this study provides valuable insights into characteristics of the health system of the country that could have identified steps and can contribute to a further reduction in abortion-related morbidity and mortality. Findings from the study suggests following recommendations:

- Further research on scaling up the SMRAC model in both intervention and comparison areas in several districts of Bangladesh can be designed and implemented to improve the quality of record keeping and data availability.
- Supportive supervision regarding use of the SMRAC model can be encouraged at the facility level, while capacity building interventions to train in statistical analysis or providing statistical support may improve sustainability of this progress. Translation of all monitoring and evaluation tools into local language (Bengali) may be a particularly important step.
- Enhanced collaboration among the health and FP wings in the public sector, and between public, private, and NGO sectors could facilitate the implementation of improved post-procedure contraception service delivery, particularly for abortion complication patients.
- Use of evidence-based, appropriate technology for all procedures particularly for abortion complication patients should be ensured. Targeted interventions to modulate facility preferences may be particularly productive toward this end.
- Service providers need to be motivated to achieve the required standards of the SMRAC Model. The Pay for Performance (P4P) model that consists of performance based incentives [21] could have the potential to entice service providers to perform to reach targets within a stipulated time.
- Quality assurance groups (QAGs) including specialists from Medical College Hospital or District Hospital and representative of professional body need to be formed to assess service providers’ performance in terms of quality and quantity as well as provide supportive feedback to motivate staff to reach SMRAC recommended levels of performance [21].

By systematic implementation of the SMRAC model, health systems can track and measure progress and gaps in their implementation and identify strategies for further reduction of abortion-related morbidity and mortality in Bangladesh.

## Abbreviations

MR: menstrual regulation
FP: family planning
PAC: post abortion care
SMRAC: safe menstrual regulation and abortion care
MVA: manual vacuum aspiration
MDG: millenium development goal
EmOC: emergency obstetric care
UN: united nations
MoH&FW: ministry of health and family welfare
DGHS: directorate general of health services
DGFP: directorate general of family planning
EPR: emergency preparedness response programme
SAC: safe abortion care
MCWC: maternal and child welfare centre
UHC: upazilla health complex
UH&FWC: upazilla health and family welfare centre
FWC: family welfare centre
NGO: non-governmental organization
IBM: international business machine
SPSS: statistical package for the social science
icddr,b: international centre for diarrhoeal disease research, Bangladesh
IRB: institutional review board
D&C: dilatation and curettage
RHSTEP: reproductive health services training and education program
WHO: world health organization
MMR: maternal mortality ratio
P4P: pay for performance
QAG: quality assurance group
